# Lifetime Traumatic Experiences and Disordered Eating among University Students: The Role of Posttraumatic Stress Symptoms

**DOI:** 10.1155/2018/9814358

**Published:** 2018-01-18

**Authors:** Vilija Malinauskiene, Romualdas Malinauskas

**Affiliations:** ^1^Department of Population Studies, Lithuanian University of Health Sciences, Kaunas, Lithuania; ^2^Department of Health, Physical and Social Education, Lithuanian Sports University, Kaunas, Lithuania

## Abstract

The associations between lifetime traumatic events (TEs), posttraumatic stress (PTS) symptoms, and disordered eating (DE) were studied in a sample of 614 university students (mean age 20 years). An anonymous questionnaire included 32 lifetime TEs, IES-revised measured PTS symptoms, and EAT-26 evaluated DE symptoms. Statistical analyses included Pearson correlations and structural equation models (SEM) with bootstrapping method. Findings reveal the prevalence of DE in 8.1% of participants, while 73.9% of students experienced at least one lifetime TE. 52.0% of students with DE had PTS symptoms (*p* < 0.0001) and 30.8% of students with lifetime TEs had PTS symptoms (*p* < 0.001). In SEM, direct paths from lifetime TEs to PTS symptoms (0.38, *p* < 0.0001) and from PTS symptoms to DE (0.40, *p* < 0.0001) were observed. The final SEM confirmed the mediating role of PTS symptoms in the path between some TEs (traffic accident and seriously injured) and DE among the university students. If PTS symptoms* are associated with DE*, then addressing PTS symptoms in the context of DE treatment may improve treatment efficacy.

## 1. Introduction

Disordered eating (DE) refers to troublesome eating behaviors, such as restrictive dieting and bingeing, which occur less frequently or are less severe than those to meet the full criteria for the diagnosis of eating disorder, and is considered as an early warning sign of an eating disorder [1]. Recent research has shown that DE is generally defined as a psychological illness [2]. Exposure to traumatic events (TEs) may be a risk factor for subsequent development of DE [3], as it can provide short-term, but not long-term, relief from trauma-related negative effects [4]. Studies investigating patients with anorexia or bulimia nervosa found high prevalence of posttraumatic stress (PTS) symptoms among them [5]. Severe long-term impairment related to the trauma in the form of posttraumatic stress disorder (PTSD) has been suggested as a possible mediator between the TEs and DE [6, 7]. Research focused on psychological state demonstrated that pathological response to trauma through PTSD or depression could be associated with DE rather than trauma exposure per se [8, 9]. In individuals with PTSD, eating disorder symptoms may be used as a means to distract from or to cope with reminders of the trauma [6].

TE is described in the DSM-5 as a situation which involves exposure to actual or threatened death, serious injury, and actual or threatened sexual violence [10]. Exposure to a TE is a required stressor criterion for a PTSD diagnosis. Additional criteria include intrusive symptoms, that is, when the trauma is reexperienced in a sudden and involuntary manner, avoidance of reminders of the trauma, and hyperarousal. PTSD is a clinical diagnosis based on strict criteria of DSM-5, while in epidemiological studies PTS symptoms are measured by structured questionnaires. The mediation pathway from trauma via PTSD to DE has been suggested previously [6], thus, traumatic exposure may influence DE both directly and indirectly via PTSD [11].

The majority of studies conducted on trauma exposure and DE have taken into account childhood TEs (emotional abuse, physical abuse, sexual abuse, emotional neglect, and physical neglect) [12–14]. Results revealed that DE patients had experienced a higher frequency of different traumas, childhood adversities in particular. Such findings are consistently reported in the extent literature, especially in relation to childhood sexual abuse, and findings suggest that it may be a strong predictor of the development of DE [15]. The associations between childhood emotional abuse and DE have also been studied [16]. Limited research has investigated adult-onset traumas [6] or lifetime TE [17]. In our study, we included a set of lifetime TEs and investigated their associations with PTS symptoms and DE.

The associations between PTSD and DE have been studied within eating disorder populations [9, 11], in a military sample [18], and in a national representative sample [19]. Studies among university students did not include PTS symptoms in their design. Effects of multiple forms of childhood abuse and sexual assault on eating disorders among university students have been investigated [13, 14, 20], but possible mediation by PTSD has not been featured as a research topic among university students. Effects of sociocultural influences and biopsychosocial factors on DE through psychological distress have been shown [21–23]. This is the first study investigating the associations between lifetime TE and DE via PTS symptoms among university students.

The objectives of the present study were to investigate the prevalence of DE, lifetime ETs, and PTS symptoms among university students in Kaunas, Lithuania. University students appear to be a specific population with elevated stress levels due to intensive daily learning activities and streaming in high academic performance. Therefore, the research, directed towards revealing other psychological adversities such as PTS symptoms and DE, is of great importance. Assuming that TEs have been associated with PTS symptoms and DE in trauma-exposed populations, we hypothesized that similar associations might be found among university students, suggesting that PTS symptoms resulting from a wide range of lifetime TEs may be associated with DE. The second objective was to assess if a wide range of lifetime TEs were associated with DE directly or through PTS symptoms. Thirdly, we tested if some specific lifetime TEs were associated with PTS symptoms and DE. We investigated the effects of sex, alcohol, substance usage, and living conditions on those associations. A multivariate design and structural equation modelling were employed to test the direct and indirect (through PTS symptoms) paths between lifetime TEs and DE. We hypothesized that lifetime TEs might be associated with DE through PTS symptoms among university students. On the other hand, we tested if some specific TEs might have a relationship with DE. Taking into account the fact that traumatic exposure might appear on momentary (e.g., “near drowning,” “seriously injured,” and traffic accident) or a repetitive basis (childhood emotional abuse or neglect), we assumed that there might be some differences in the associations between the specific TEs and DE.

## 2. Methods

### 2.1. Participants and Procedure

We employed a multistage sampling method whereby four universities in Kaunas, Lithuania, were randomly selected in the spring semester of the 2012-2013 academic years. Two groups were randomly selected from each faculty (health, social, and natural sciences). The sample size comprised 772 students and they were invited to participate in the study during lecture time. Participation in this study was fully voluntary. 83 students did not participate in selections; nobody refused to answer the questionnaire. Participants were 689 students who were enrolled in health (41.3%), social (31.0%), and natural sciences (27.7%) courses. After selection of fully completed questionnaires* (EAT-26, IES-revised, questionnaire on lifetime TEs*), 614 students (182 males, 432 females) were investigated (response rate 79.53%). Ages ranged from 18 to 35 years (M = 20.74, SD = 1.69).

Questionnaires were completed during lecture time and then collected on the spot. Students were informed that participation was voluntary, that their responses would remain anonymous, and the respondents were provided with sweets. They all provided written informed consent before completing the measures. The research protocol was approved by the Bioethics Centre of the University of Health Sciences.

### 2.2. Lifetime Traumatic Experiences

Participants were presented with a list of lifetime TEs, with good psychometric properties tested in Scandinavian and Baltic populations [24]. Previous studies have demonstrated that the included events are frequently experienced by youths across nations and cultures and that these events may be traumatizing [24–29]. These events were related to experiences that occurred during adolescence or early adulthood. Participants were asked if they (direct) or witnessing an event or having any of their family members (indirect) experienced the following events: traffic accident, other serious accident (fire, explosion, etc.), physical assault, rape, being injured, threats of being beaten, near drowning, attempted suicide, robbery/theft, pregnancy/abortion, serious illness, death of someone close, divorce, humiliation or persecution by others (bullying), absence of a parent, and other traumas. Only direct exposures were evaluated in this study as potentials for eating disorders.

Childhood maltreatment was assessed retrospectively by standardized self-report questionnaire [30] that included 20 items that were indicative of an abuse domain: sexual abuse (3 items), physical abuse (5 items), emotional abuse (6 items), and physical neglect (6 items). For example, “parents/guardians have through their behavior shown that you were unwanted, unloved, and worthless.” All responses were answered “yes” or “no” (yes = 1; no = 0) in relation to whether the events described occurred or not.

The trauma index was calculated by summing up all lifetime TEs (yes/no) to which the participants had been exposed over the course of their life (M = 2.05, SD = 2.33).

### 2.3. Posttraumatic Stress Symptoms

The Impact of Event Scale-Revised (IES-R) [31] was used to assess PTS symptoms following lifetime TEs. The IES-R has 22 items; these assess hyperarousal symptoms such as anger and irritability, heightened startle response, difficulty in concentrating, and hypervigilance; the intrusion scale assesses a dissociative-like reexperience and true flashbacks. Eight items are used to assess avoidance. Respondents were asked to rate each item according to the past seven days. Scoring of the total scale over 33 was considered as a cut-off for a “probable PTSD case.” This scale was translated into Lithuanian and cultural adaptation was performed [32]. Internal consistency for the total IES-R scale was high in the present study (Cronbach's alpha = 0.95). Cronbach's alpha for the intrusion subscale was 0.91, for avoidance 0.87, and for hyperarousal 0.87.

### 2.4. Eating Attitudes Test-26

The Eating Attitudes Test-26 (EAT-26) is a 26-item scale used to assess DE risk based on attitudes, feelings, and behaviors related to eating and eating disorder symptoms [33]. It consists of three subscales which are dieting, bulimia and food preoccupation, and oral control. Using a six-point Likert scale, they reported how often 26 statements about DE attitudes and behaviors were true for them. The dieting subscale measured dieting behaviors and the drive for thinness. The bulimia and food preoccupation subscale tapped binge eating and vomiting. The oral control subscale assessed perceived social pressure to gain weight. Items in the scale are rated on a six-point Likert scale: always (1), usually (2), often (3), sometimes (4), rarely (5), and never (6). For all items except item 26, the responses “sometimes,” “rarely,” and “never” receive a score of 0 and the responses “always,” “usually,” and “often” receive scores of 3, 2, and 1, respectively. Scoring for item 26 is in the reverse manner. Scale score is the sum of all items ranging from 0 to 58. Participants who scored 20 or more were considered to have a high level of concern about dieting or problematic eating behaviors. Participants who scored less than 20 were considered to have no symptoms of DE. The score of subscales ranged in dieting from 0 to 30, bulimia and food preoccupation from 0 to 18, and oral control from 0 to 14. This scale was translated into Lithuanian and cultural adaptation was performed [34]. In the present study, the internal consistency of the EAT-26 was good (Cronbach's alpha = 0.87). Cronbach's alpha for the dieting subscale was 0.87, for the bulimia and food preoccupation subscale 0.76, and for the oral control subscale 0.77.

### 2.5. Demographics

Demographic information was collected to assess subjects' sex, age, and living conditions (living in a separate flat alone, living at a state dormitory with friends, living with parents, and living with a husband/romantic partner); smoking and alcohol/drug usage information was evaluated with single questions assessing the frequency of the exposure.

### 2.6. Statistical Analyses

Descriptive statistics were calculated in SPSS 21.0 for Windows. Differences between groups were evaluated using chi-squared tests. The correlations between the study variables: sum of the lifetime TEs as trauma index; EAT-26 scale and subscales dieting, bulimia and food preoccupation, and oral control; IES-revised scale and subscales (intrusion, avoidance, and hyperarousal), trauma index* as continuous variables* were identified using Pearson's product moment correlation.

*Structural Equation Models. *To address the primary objectives, a series of structural equation models (SEMs) assessing associations among TEs, PTS symptoms, and DE were estimated using Mplus, version 5.2. SEM has the advantage of simultaneously estimating paths among multiple constructs while modelling measurement error [35]. A series of SEMs were performed to find the best data fit to the model according to the model fit indices. The indices used to assess goodness of fit for the models included the chi-squared test of model fit with *p* value, the comparative fit index (CFI), the Tucker-Lewis index (TLI), root mean square error of approximation (RMSEA) with its 90% CI, and weighted root mean square residual (WRMR) [36].

The *z*-scores were calculated for the variables: dieting, bulimia and food preoccupation, and oral control as those variables were not normally distributed. The *z*-scores were calculated by subtracting the means from an individual raw scores and then dividing the differences by the standard deviations.

In statistics, “latent variables,” as opposed to observed variables, are variables that are not directly observed but are rather inferred (through a mathematical model) from other variables that are observed (directly measured). Mathematical models that aim to explain observed variables in terms of latent variables are called latent variable models. One advantage of using latent variables is that they can serve to reduce the dimensionality of data. A large number of observable variables can be aggregated in a model to represent an underlying concept, making it easier to understand the data. Latent refers to the fact that even though these variables were not measured directly in the research design they are the ultimate research goal. The nature of the latent variable is intrinsically related to the nature of the indicator variables used to define them. In the most usual case, we structure the model so that the indicators are “effects” of the latent variable, like in the case of the common factor analysis [37].

Observed dependent variables included continuous (EAT-26 subscales dieting, bulimia and food preoccupation, and oral control; trauma index) and binary and ordinal (IES-revised intrusion and avoidance subscales) variables. The hyperarousal subscale was excluded from the analysis as it correlated extremely with the other two subscales of PTS symptoms (intrusion and avoidance) and provided a model which did not fit to the data. Continuous latent variables were PTS symptoms and DE (EAT-26 total scale). The intercorrelation effects of the dieting and bulimia subscales of EAT-26 were evaluated in the models. The observed independent variable was sex. The models were estimated controlling for sex as this correlated with PTS symptoms and some TEs and showed a good fit for the model characteristics. Models were controlled for alcohol and drug usage as well as socioeconomic data (living conditions), but these solutions did not provide a data fit to the models.

**Figure 1:**
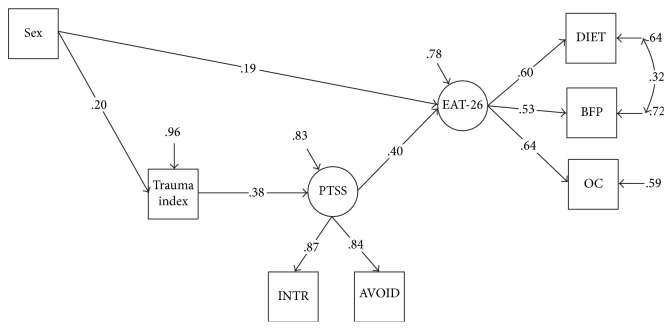
Structural model of the relationship between trauma index, PTS symptoms, and DE.* Note.* PTSS: posttraumatic stress symptoms; INTR: Intrusion; AVOID: Avoidance; EAT-26: DE; DIET: dieting; BFP: bulimia and food preoccupation: OC: oral control.

**Figure 2:**
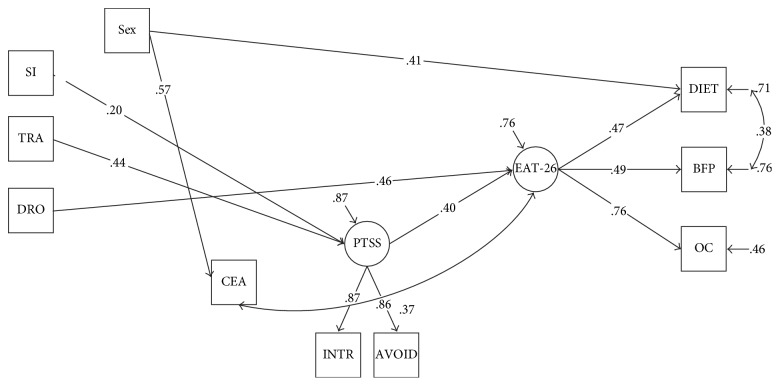
Final structural model of the relationship between specific TEs, PTS symptoms, and DE.* Note.* SI: seriously injured; TRA: traffic accident; DRO: near drowning; CEA: childhood emotional abuse; PTSS: posttraumatic stress symptoms; INTR: Intrusion; AVOID: Avoidance; EAT-26: DE; DIET: dieting; BFP: bulimia and food preoccupation; OC: oral control.

*All direct paths in the diagrams were calculated, but only significant paths are presented in the diagrams (Figures 1 and 2).*

Model I was performed using all TEs as trauma index. To statistically evaluate the adequacy of the hypothetical model for the empirical data, multiple goodness-of-fit indices were used. Direct (sex and sum of lifetime TEs as trauma index) effect on EAT-26 and indirect effect of the trauma index through PTS symptoms on EAT-26 were tested using the bootstrapping technique. Bootstrapping is a recommended analytic technique that involves repeated random sampling observations with replacement from the dataset [36]. In fact, the bootstrap method has been shown to be more accurate than traditional methods in many circumstances. A great advantage of bootstrap is its simplicity. It is a straightforward way to derive estimates of standard errors and confidence intervals for complex estimators of complex parameters of the distribution, such as percentile points, proportions, odds ratio, and correlation coefficients. Bootstrap is also an appropriate way to control and check the stability of the results [36]. The use of conventional tests of significance for testing indirect effects can be problematic in practice, because the product of two or more regression coefficients is tested. This product often is not normally distributed, which can make conventional tests of significance unreliable [38] and therefore recommend the use of asymmetric confidence intervals based on bootstrap methods for significance testing of indirect effects as a more appropriate alternative.

In Model II, specific TEs were included as binary variables. Before starting with the model, correlations between specific TEs were calculated. A series of SEMs were performed to find the best data fit to the model according to the model fit indices. Finally, those TEs that were significantly associated with PTS symptoms or DE and showed good fit to the model indices were included in Model II (“traffic accident,” “seriously injured,” “near drowning,” and “childhood emotional abuse”). Direct (sex and specific lifetime TEs) effect on PTS symptoms and EAT-26 and indirect effects of specific lifetime TEs through PTS symptoms on EAT-26 were tested.

We used 5,000 95% bootstrap confidence intervals in order to test the significance of indirect effects from TEs on DE via PTS symptoms. An indirect effect was considered to be significant at the .05 level if the 95% CI from 5,000 bootstrap samples does not include zero.

## 3. Results

### 3.1. Descriptive Statistics

About 32.2% of the students lived in a state dormitory:* 28.7% smoked, 87.9% consumed alcohol, and 11.1% consumed drugs.* At least one lifetime trauma had been experienced by 73.9% of students; the prevalence of PTS symptoms was 27.4% and DE 8.1% (Table 1). Forty-four per cent of students had suffered more than one trauma during their lifespan. Fifty-two per cent of students with DE had PTS symptoms (*p* < 0.0001) and 30.8% of students with lifetime TEs had PTS symptoms (*p* < 0.001). Chi-squared tests showed sex differences in PTS symptoms prevalence (132 (30.6%) of females and 36 (19.8%) of males had PTS symptoms; *p* = 0.006) as well as prevalence of lifetime TEs (340 (78.7%) of females and 114 (62.6%) of males experiences had experienced at least one traumatic event; *p* < 0.0001). No sex differences in the prevalence of DE were found. In our sample, the most common TEs were involved in traffic accident (126, 20.5%), death of some close (132, 21.5%), divorce (76, 12.4%), near drowning (84, 13.7%), seriously injured (48, 7.8%) serious illness (80, 13.0%), other serious accident (76, 12.4%), and childhood emotional abuse (84, 13.7%). In this study, significant differences were found between rape (*N* = 10 (1.6%)) and sexual harassment (*N* = 14 (2.3%)) and PTS symptoms and DE (*p* < 0.0001); the prevalence of childhood physical abuse in this study was 1.6% and neglect 2.0 per cent. Due to the small number of cases, those TEs did not fit well with the model indices and were not included in SEMs.

Table 2 presents Pearson correlations between the study variables, indicating that trauma index, PTS symptom scale and subscales, and EAT-26 scale and subscales correlate. The majority of the variables were found to be significantly and positively correlated.

### 3.2. SEM Results

Lifetime TEs, PTS symptoms, DE, and sex as variables were analysed simultaneously in SEM. Model I and the hypothesized model provided a good fit to the data (chi-squared = 16.437, degrees of freedom = 10, *p* value = 0.088, CFI = 0.98, TLI = 0.96, RMSEA = 0.043, 90% CI 0.000–0.079, WRMR = 0.56) (Figure 1). All direct paths in the diagrams were calculated, but only significant paths are presented in Figures 1 and 2.

In Model I, we found direct paths from trauma index to PTS symptoms and DE and from sex to trauma index and DE in SEM (Figure 1). The structural paths were significant, with the exception of the path from trauma index to DE and from sex to PTS symptoms. Direct paths from lifetime TEs to PTS symptoms (0.38, *p* < 0.0001) and from PTS symptoms to DE (0.40, *p* < 0.0001) were observed. Then we used 5,000 95% bootstrap confidence intervals to test the significance of indirect effects from lifetime TEs to DE via PTS symptoms. The indirect effect of trauma index through PTS symptoms to DE (EAT-26) was confirmed (0.156, 95% CI 0.016–0.0670) (does not include zero).

Variables that are placed on the model diagrams (Figures 1 and 2) are either observed variables or latent variables. Observed variables exist in our data file and are represented in the model diagram by rectangular (square) boxes. Latent variables do not exist in the data but are unobserved variables that are thought to underlie some subset of the observed variables. Latent variables are represented by the circular (oval) shapes.

Furthermore, many models were estimated with different traumatic event combinations and the solution in Model II provided a good fit to the data (chi-squared = 33.156, degrees of freedom = 23, *p* value = 0.078, CFI = 0.97, TLI = 0.95, RMSEA = 0.036, 90% CI 0.00–0.061, WRMR = 0.66) (Figure 2).

In Model II, some specific TEs were included as binary variables (involved in traffic accident, seriously injured, near drowning, and childhood emotional abuse) and again the direct (from specific TEs to PTS symptoms and DE) and indirect paths from specific TEs to DE via PTS symptoms were tested (Figure 2). We found significant direct paths from “traffic accident” (0.44, *p* = 0.004) and “seriously injured” (0.20, *p* = 0.003) to PTS symptoms and from PTS symptoms to DE (0.40, *p* < 0.0001). We found a significant direct path from “near drowning” to DE (0.46, *p* = 0.043), and an indirect effect of “traffic accident” to DE via PTS symptoms was confirmed (0.129, 95% CI 0.032–0.303) (does not include zero). The indirect effect of “seriously injured” to DE via PTS symptoms was also confirmed (0.208, 95% CI 0.058–0.460) (does not include zero). We found a correlational relationship between childhood emotional abuse and DE (0.37, *p* < 0.01) in this sample of university students.

## 4. Discussion

The etiology of DE is multifactorial; however, there is a compelling body of research suggesting that stressful life events are implicated in the onset and maintenance of DE [8, 16, 20]. This study aimed to assess the prevalence of lifetime TEs, PTS symptoms, and DE in a sample of university students in Lithuania and to evaluate the associations between those variables, employing SEM. It was the first study on this topic not only in Lithuania but also among university students as a whole. An important peculiarity of SEM is that its primary objective is to check the validity of all models, while the hypotheses about the specific relationships among variables are checked later. Another peculiarity is that the regressive relationships in the model are understood as causative relationships. Though SEM alone cannot “prove” that a certain variable in a certain relationship is a cause and another variable is a consequence, it allows checking how well a certain model of causative factors matches the available empirical data [36].

We found a definite high prevalence of lifetime TEs (73.9% of students experienced at least one lifetime trauma), while other studies also found a high prevalence of TEs ranging from 70% [39] to 86% [28].

The presence of PTS symptoms and the association between trauma exposure and subsequent PTS symptoms was found in other studies among university students [40, 41], indicating no differences when compared to the general population. Sex differences in PTSD prevalence were found among other populations as well as in our study, indicating that girls suffered more often than boys [28], though it was not confirmed in SEM. The prevalence of DE in other studies did not differ substantially from this study, being 11.5 among undergraduate students in the USA [20]. In our study, prevalence of PTS symptoms in the total sample is relatively high but does not differ substantially from the subgroup of students with lifetime traumatic experience (27.4% versus 30.8%), underlying the notion that TEs generally stronger associated with PTSD (e.g., sexual and physical abuse) are rarely represented by the sample of general population (e.g., students).

### 4.1. Traumatic Experiences and Disordered Eating

This study provides further support for an indirect relationship between TEs and eating psychopathology. Taken together, our results suggest that lifetime TEs are significantly associated with PTS symptoms and PTS symptoms with DE, while PTS symptoms mediated the association between lifetime TEs and DE. We did not find a direct path between trauma index and DE. Other studies also confirmed that severe long-term impairment related to trauma in the form of PTSD has been suggested as a possible mediator between a TE and DE [6, 42].

### 4.2. PTS Symptoms and Disordered Eating

In our study we had no possibility of diagnosing PTSD clinically, but, as usual in epidemiological studies, we measured PTS symptoms and found the direct path with DE and another finding was that PTS symptoms mediated the associations between TE and DE.

As PTSD is characterized by intrusive memories, images, and/or nightmares about TEs, avoidance of distressing memories, trauma reminders, and thoughts and feelings about TEs, and DE behaviors such as dieting, binge eating, and purging has the strong potential to facilitate escape and avoidance of distressing memories, thoughts, and feelings and to decrease the hyperarousal symptoms of PTSD [43]. Any degree of successful avoidance of trauma-related thoughts, feelings, and memories is reinforcing and promotes maintenance of the DE. The psychological impact associated with experiencing a trauma at an earlier stage of life is said to persist long into adulthood. On average, a diagnosis of PTSD occurs during early adulthood [44] with persisting memories of TEs and the need to cope with them. The individual is trying to avoid or regulate PTSD symptoms, further maintaining atypical eating behaviors through maladaptive coping [45]. Attempts to avoid memories of TEs increase the likelihood of developing a range of psychological difficulties, including eating disorders. Therefore, DE might be a maladaptive method of coping with PTSD symptoms [45].

### 4.3. Types of Trauma and Disordered Eating

Data from other studies in Lithuania confirm that “traffic accident” and “near drowning” are important TEs in the context of lifetime trauma history [26, 28]. We assessed the role of the most frequent types of lifetime traumatic experience in SEM (traffic accident, seriously injured, near drowning, and childhood emotional abuse) in the associations with DE. In the present study, when variables were analysed simultaneously in SEM, we found a direct path between “near drowning” and DE; a correlational relationship was found between childhood emotional abuse and DE. It is a new finding in our study that “near drowning” is directly associated with DE. Recent research suggests that different brain areas are particularly susceptible to different types of trauma [46]. Literature analysis confirms that drowning is a specific traumatic event with probable brain injury. The neuropathological consequences of drowning have not been fully determined, yet MR imaging showed functional brain changes such as reduced brain reserve in motor and visual brain regions in victims of moderate drowning [47] and another study showed damage to the paediatric anoxic brain after drowning, predominantly the posterior limbs of the internal capsule [48]. Recent scientific knowledge is unable to answer the question of why youths after drowning have DE and which brain parts are responsible; yet first studies of structural brain imaging among DE patients have provided evidence that brain reward circuits may be altered in patients with anorexia or bulimia nervosa [49].

Another finding in our study is the bidirectional relationship (covariance) between childhood emotional abuse and DE. The definitions have tended to conceptualize childhood emotional abuse as being on a continuum, with the repetitive, sustained nature of the acts being a crucial defining feature. The definition of childhood emotional abuse as “the sustained, repetitive, inappropriate emotional response to the child's experience of emotion and its accompanying expressive behavior” [50, p. 456] appears to contain most of the key components of what we understand to comprise childhood emotional abuse. Childhood emotional abuse may lead to immediate consequences (e.g., withdrawal, academic underachievement, and emotional instability), but it has serious long-term effects upon human functioning [51]. Acting on a repetitious basis, childhood emotional abuse may gain a bidirectional relationship with DE. It is possible that students with eating disorders lasting in overweight or obesity might be emotionally abused in earlier periods of their life, and vice versa: they might develop DE due to exposure to childhood emotional abuse. This is observational study and the causality cannot be determined, though SEM enables revealing covariances between variables that differ in time.

It is interesting to note that most studies investigating the association between childhood emotional abuse and eating psychopathology do not include PTS symptoms in their design [12, 20, 51] and indicate that childhood emotional abuse is the most associated with eating disorder pathology in adulthood [8, 16, 20]. On the other hand, recent research analysis confirmed that childhood adversities were most strongly and consistently associated with PTSD [52] and childhood emotional abuse was a strong predictor of PTSD in adulthood [53]. It could be questioned why in our study in Model II childhood emotional abuse was not directly associated with PTS symptoms. Probably, the answer might be that different TEs act in different ways in the association between DE and PTS symptoms. This could be a topic for future research.

Other TEs, like “traffic accident” or being “seriously injured,” were found to be directly associated with PTS symptoms, and an indirect effect on DE via PTS was confirmed. The associations between TEs and PTS symptoms were confirmed in other studies [28, 54], but further associations of different TEs with DE might be a topic for future research.

### 4.4. Study Limitations

Several limitations of the present study should be noted. Firstly, the findings may not be generalized to the whole population of university students in Lithuania. A different sample might be employed in further investigations. Secondly, a causal relationship could not be established due to the nature of the cross-sectional study design used in this study. A longitudinal or prospective study is required to further confirm and test the pathways found in this study. The results in our study do not imply that these are the only valid models for the development of DE in university students, although the models hypothesized in this study provided a good fit to the data [36]. Other forms of childhood maltreatment, such as physical abuse, emotional and physical neglect, and sexual abuse and harassment, due to the small number of cases, did not fit for SEMs. Further research should investigate other possible influences and specific TEs in the development of DE among university students, as well as studies directed towards targeted populations (e.g., physically and sexually abused). There might be other factors which may influence DE, such as biological and psychological factors, acting directly or through PTS symptoms, which were not examined in our study due to limitations in terms of time and financial resources.

Another limitation of the study is the fact that the time of onset of PTS symptoms and DE symptoms as well as age of lifetime TEs occurrence was not included into the questionnaire, though in this case the recall bias might not be avoided. Also, the physical (thyroid, adrenal, and metabolism disorders associated with obesity) and mental (anxiety, depression, and sleeping disorders) comorbidities or social conditions (marriage, divorce, pregnancy, children, and employment) were not considered at all that could be associated with psychological stress and consequently disordered eating. The help-seeking history and if the subjects received any treatment for PTS symptoms and/or disordered eating were not considered.

## 5. Conclusions

This study confirmed that lifetime TEs were associated with DE via PTS symptoms. The final SEM confirmed a direct path between near drowning and DE, a correlational relationship between childhood emotional abuse and DE, and the mediating role of PTS symptoms in the path between some TEs (traffic accident and seriously injured) and DE among the university students.

Our study has practical implications. If PTSD is* associated factor with* DE symptoms, then addressing PTSD in the context of DE treatment may improve treatment efficacy. In intervention studies, the trauma history revealing the specific TEs and PTSD or its symptoms must be expressly and satisfactorily addressed in order to facilitate full recovery from the DE and associated psychopathology. The new finding in our study was that specific TEs act in different ways in the path with DE that requires future investigation.

## Figures and Tables

**Table 1:** Associations between TEs, PTS symptoms, and DE.

	Total		PTS symptoms			DE		
	*N*	%	*N*	%	*p*	*N*	%	*p*
DE								
No	564	91.9						
Yes	50	8.1						
PTS symptoms								
No	446	72.6				24	48.0	
Yes	168	27.4				26	52.0	<0.0001
Sex								
Boys	182	29.6	36	19.8		10	5.5	
Girls	432	70.4	132	30.6	0.006	40	9.3	0.119
Lifetime TEs								
No	160	26.1	28	17.5		10	6.3	
Yes	454	73.9	140	30.8	<0.001	40	8.8	0.309
Traffic accident	126	20.5	50	39.7	<0.001	12	9.5	0.525
Other serious accident	76	12.4	26	34.2	0.152	10	13.2	0.088
Threats of being beaten	40	6.5	16	40.0	0.064	4	10.0	0.567
Pregnancy/abortion	14	2.3	8	57.1	0.011	0	0	0.260
Physical assault	50	8.1	22	44.0	0.006	10	20.0	<0.001
Absence of a parent	90	14.7	40	44.4	<0.0001	8	8.9	0.780
Seriously injured	48	7.8	30	62.5	<0.0001	6	12.5	0.250
Death of someone close	132	21.5	32	24.2	0.364	14	10.6	0.243
Serious illness	80	13.0	36	45.0	<0.0001	6	7.5	0.822
Rape	10	1.6	10	100.0	<0.0001	6	60.0	<0.0001
Sexual harassment	14	2.3	12	85.7	<0.0001	6	42.9	<0.0001
Divorce	76	12.4	38	50.0	<0.0001	2	2.6	0.061
Attempted suicide	16	2.6	8	50.0	0.040	0	0	0.228
Witnessed other people being injured or killed	18	2.9	10	55.6	0.006	2	11.1	0.640
Bullying	14	2.3	4	28.6	0.918	4	28.6	<0.005
Near drowning	84	13.7	30	35.7	0.065	16	19.0	<0.0001
Robbery/theft	28	4.6	8	28.6	0.883	6	21.4	0.009
Personal serious health problem	44	7.2	22	50.0	<0.0001	6	13.6	0.167
Childhood emotional abuse	84	13.7	34	40.5	0.004	10	11.9	0.175
Childhood physical abuse	10	1.6	2	20.0	0.599	2	20.0	0.167
Severe childhood neglect	12	2.0	8	66.7	0.002	4	33.3	<0.001

*Note*. *p* calculated in chi-squared test; PTS symptoms: posttraumatic stress symptoms; DE: disordered eating; TEs: traumatic events.

**Table 2:** Pearson correlations among study variables.

	Mean (SD)	1	2	3	4	5	6	7	8	9	10
(1) Sex		1									
(2) EAT-26	36.47 (17.09)	.26^*∗*^	1								
(3) PTSS	22.63 (18.25)	.10^*∗*^	.30^*∗∗*^	1							
(4) Trauma index	2.05 (2.33)	.21^*∗∗*^	.20^*∗∗*^	.38^*∗∗*^	1						
(5) Dieting	19.96 (11.20)	.31^*∗∗*^	.92^*∗∗*^	.23^*∗∗*^	.19^*∗∗*^	1					
(6) Bulimia	6.74 (4.79)	.04	.73^*∗∗*^	.24^*∗∗*^	.13^*∗∗*^	.54^*∗∗*^	1				
(7) Oral control	9.77 (5.13)	.16^*∗∗*^	.64^*∗∗*^	.29^*∗∗*^	.15^*∗∗*^	.38^*∗∗*^	.33^*∗∗*^	1			
(8) Intrusion	7.71 (6.92)	.09^*∗*^	.28^*∗∗*^	.94^*∗∗*^	.35^*∗∗*^	.20^*∗∗*^	.24^*∗∗*^	.28^*∗∗*^	1		
(9) Avoidance	9.46 (7.51)	.11^*∗∗*^	.29^*∗∗*^	.92^*∗∗*^	.34^*∗∗*^	.22^*∗∗*^	.21^*∗∗*^	.28^*∗∗*^	.78^*∗∗*^	1	
(10) Hyperarousal	5.45 (5.21)	.08	.26^*∗∗*^	.92^*∗∗*^	.37^*∗∗*^	.21^*∗∗*^	.20^*∗∗*^	.22^*∗∗*^	.85^*∗∗*^	.76^*∗∗*^	1

*Note.* ^*∗*^ *p* < .05; ^*∗∗*^*p* < .01; EAT-26: Eating Attitudes Test-26; PTSS: posttraumatic stress symptoms; sex was included as dummy variable.
